# The Role of Mandibular Thickness in Determining Anteroposterior Skeletal Relationships

**DOI:** 10.3390/dj13010003

**Published:** 2024-12-24

**Authors:** Akihiro Tsuboi, So Koizumi, Masahiro Takahashi, Yu Hikita, Tetsutaro Yamaguchi

**Affiliations:** Department of Orthodontics, School of Dentistry, Kanagawa Dental University, Yokosuka 238-8580, Japant.yamaguchi@kdu.ac.jp (T.Y.)

**Keywords:** anteroposterior skeletal relationship, computed tomography, craniofacial growth, mandibular morphology, mandibular growth

## Abstract

**Objectives:** This study aimed to explore the association between the ANB angle and mandibular bone thickness in individuals with skeletal malocclusion. **Methods:** We analyzed 224 adults, with a gender imbalance noted (68 men and 156 women), and an age range between 18.0 and 65.1 years. The thicknesses of the ramus (RT) and the body of the mandible (BT) were measured at 25 sites on each side (left and right) of the jaw, using CT images. The main focus was on the statistical associations between the ANB angle and the ratio (BT-RT)/volume of the mandible (V). **Results:** Results revealed a significant negative association between (BT-RT)/V and the ANB angle (*p* < 0.001), while a positive association was found for RT/V (*p* < 0.001). No significant association emerged for BT/V. **Conclusions:** The findings indicated that with increased mandibular protrusion, the ramus thickness decreased, whereas the body thickness remained unchanged. The differing relationships in mandibular thickness underscore the significance of CT imaging in predicting craniofacial growth patterns, particularly in observing discrepancies between the mandibular body and ramus as they relate to the anterior–posterior jaw relationship.

## 1. Introduction

The mandible consists of the ramus and the body and is a movable structure that has two condylar processes. At the base of the condylar process, the lateral pterygoid muscle attaches, while the temporalis muscle attaches to the coronoid process located anteriorly to the condylar process [1].

Controlling the anteroposterior growth of the jaw presents a challenge despite the existence of devices, such as face masks, activators, and chin caps, aimed at achieving and maintaining therapeutic goals [2,3]. The mandible, one of the anatomical structures of the craniofacial region, experiences its growth peak last. Improvements made during orthodontic treatment in the mixed dentition period can influence future craniofacial morphology, thereby aiding in clarifying treatment objectives and planning [4]. Additionally, the effectiveness of such treatment is crucial in determining the necessity of future surgical orthodontic interventions, necessitating accurate growth predictions [5].

Recently, incorporating three-dimensional information from cone-beam computed tomography (CBCT) has become increasingly common [6]. Although sporadic, studies investigating the relationship between skeletal patterns and the three-dimensional morphology of the mandible have been reported [7,8]. Accurate prediction of craniofacial growth solely from two-dimensional images remains challenging, emphasizing the necessity of investigating three-dimensional characteristics using CBCT for more precise growth prediction [9].

The anteroposterior skeletal relationship influences the course and length of the masticatory muscles, thereby affecting the mechanical advantage in bite force [10,11]. Thus, differences in the anteroposterior skeletal relationship may lead to varying effects of the masticatory muscles on the mandible, potentially leading to differences in mandibular thickness.

Previous studies using CBCT for three-dimensional measurements have compared the thickness of the mandibular lingula between skeletal Class II and III malocclusions, as well as the thickness of the ramus (RT) on the non-deviated and deviated sides of the mandible. These studies have suggested that the ramus tends to be thinner in skeletal Class III mandibles [7,12]. Additionally, Katayama et al. [8] reported no significant difference in mandibular volume concerning the anteroposterior skeletal relationship, suggesting that while the ramus may exhibit reduced thickness in skeletal Class III malocclusion, there could be an increase in thickness elsewhere in the mandible. As highlighted by Maspero et al. [13], numerous studies on the growth patterns of the mandibular condyle, and ramus have demonstrated the critical role of the ramus in mandibular growth. However, the relationship between the growth patterns of the ramus and body of the mandible remains unclear, with no studies investigating the differences in thickness between the body of the mandible (BT) and ramus (BT-RT). Additionally, the present study considered the possibility that the mandible size could vary with physique and adopted a method of dividing the difference in thickness by the volume of the mandible [(BT-RT)/V]. Identifying new morphological features that have a causal relationship with the anteroposterior skeletal relationship is important for making more accurate growth predictions.

In this study, we aimed to examine the association between the anteroposterior skeletal relationship and the thicknesses of the body of the mandible and ramus. The null hypothesis posits that the regression coefficient for the subspinale-nasion-supramentale angle (ANB) is zero in a multiple regression analysis, in which the dependent variable is (BT-RT)/V and the independent variables are the ANB, Frankfort-mandibular plane angle (FMA), sex, age, and differences in CT equipment.

## 2. Materials and Methods

This cross-sectional study comprised a universal sampling of 224 adult patients treated at the Orthodontic Department of Kanagawa Dental University Hospital between February 2017 and March 2023. As all adult patients seen during this period were included, the results are considered representative of the adult orthodontic patient population at this hospital during that time. The sample included 68 males (mean age 26.6 years, range 18.3–60.0 years) and 156 females (mean age 29.1 years, range 18.0–65.1 years). Skeletal classification revealed 54 Class I (−1° ≤ ANB < 4°), 84 Class II (ANB ≥ 4°), and 86 Class III (ANB < −1°) patients. Based on FMA, there were 30 low-angle (FMA < 23°), 90 normal-angle (23° ≤ FMA ≤ 30°), and 108 high-angle (FMA > 30°) patients. [14]

The inclusion criteria and exclusion criteria are as follows.

Inclusion Criteria:

- Adults aged 18 years or older;
- No congenital disorders (diseases that affect the craniofacial region, such as cleft lip and palate, cleft jaw, and Down syndrome) or systemic diseases (patients with a history of trauma to the craniofacial region, cysts or tumors, periodontal disease, or diseases that are causally related to diabetes);
- No mandibular deviations (0 mm ≤ Me deviation ≤ 2 mm);
- Skeletal Class I, Class II, or Class III.

Exclusion Criteria:

- Individuals with congenital disorders;
- Individuals with systemic diseases;
- Individuals aged < 18 years;
- Individuals with mandibular deviations (2 mm < Me deviation).

The Ethics Committee of Kanagawa Dental University approved this study (approval number: 911).

### 2.1. Measurements

In this study, we predicted that the larger the mandible, the greater the thickness value. Therefore, we thought that it was necessary to eliminate the effect of mandible size in order to correctly evaluate bone thickness. We devised a method of eliminating the effect by dividing the measured thickness by the volume of the mandible. In order to evaluate the validity of the method, we set three dependent variables: (1) pure thickness measurement values, (2) thickness measurement values divided by SN, and (3) the ratio of thickness between the mandibular ramus and body, and conducted the same multiple linear regression analysis for each, in order to evaluate the validity of the thickness measurement method used in this study. The basis for setting each dependent variable is as follows. (1) This was established to evaluate whether or not the size of the mandible needs to be taken into account in the first place. (2) The SN is often used as a standard for cranial size, and it was established based on the idea that the size of the mandible is proportional to the size of the cranium to a certain extent. (3) We established this by calculating the ratio of the thickness of the mandible to the thickness of the body, as we thought that this would cancel out the effect of the size of the mandible.

**Figure 1:**
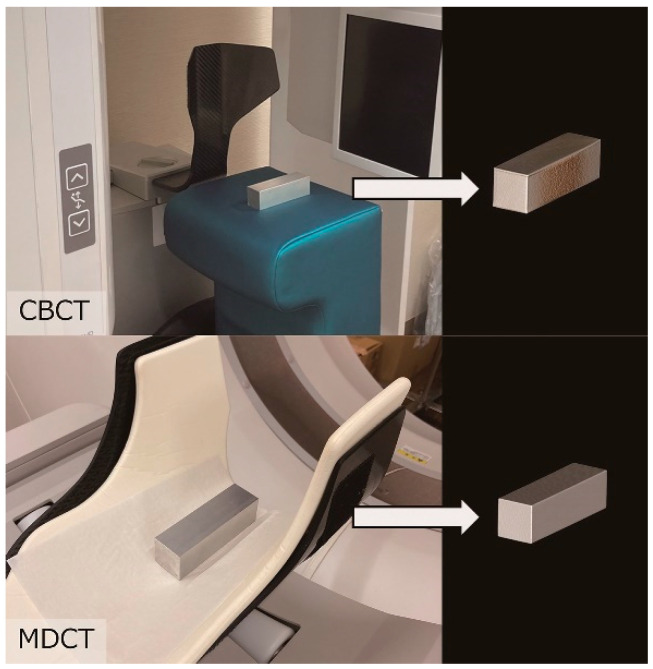
Validation of cone-beam computed tomography (CBCT) and multi-detector computed tomography (MDCT) measurement accuracy. Images show a known volume of aluminum blocks on each CT device. Three-dimensional CT images of these blocks were acquired using optimized experimental parameters.

CBCT and multi-detector computed tomography (MDCT) images were acquired using our radiology department’s equipment: CBCT (KaVo OP 3D Vision, Biberach, Germany) at 120 kV, 5 mA, a voxel size of 0.3 mm, and an acquisition time of 17.8 s; and MDCT (Aquilion Prime, Tochigi, Japan) at 120 kV, 30 mA, a voxel size of 2.0 mm, a pitch factor of 0.637, and a helical pitch of 51.0. Discrepancies between CBCT and MDCT models were assessed using the method described by Katayama et al. [8] (Figure 1), which revealed minimal discrepancies that did not affect outcomes.

**Figure 2:**
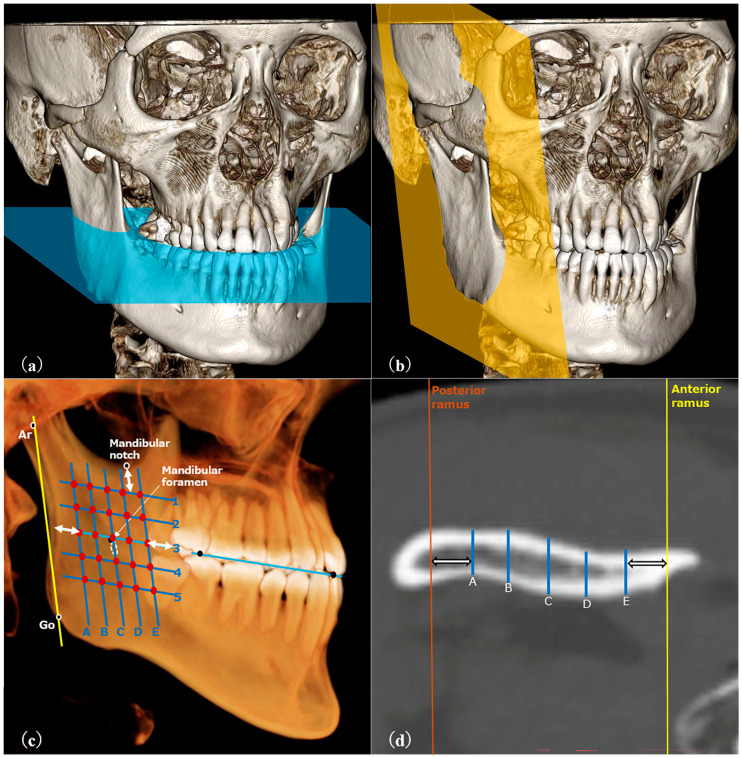
(**a**–**d**) CT images viewed with DICOM viewer. (**a**) The axial plane (blue plane) was parallel to the occlusal plane. (**b**) The para-sagittal plane (yellow plane) was parallel to the ramus. (**c**) Five oblique lines, parallel to the ramus’s posterior border and 5 mm from its surfaces, were drawn. Five evenly spaced horizontal lines intersected the middle oblique line 5 mm below the ramus’s top. Thickness was measured at 25 points to average the ramus’s thickness. (**d**) An example of measurement at a certain cross-section.

**Figure 3:**
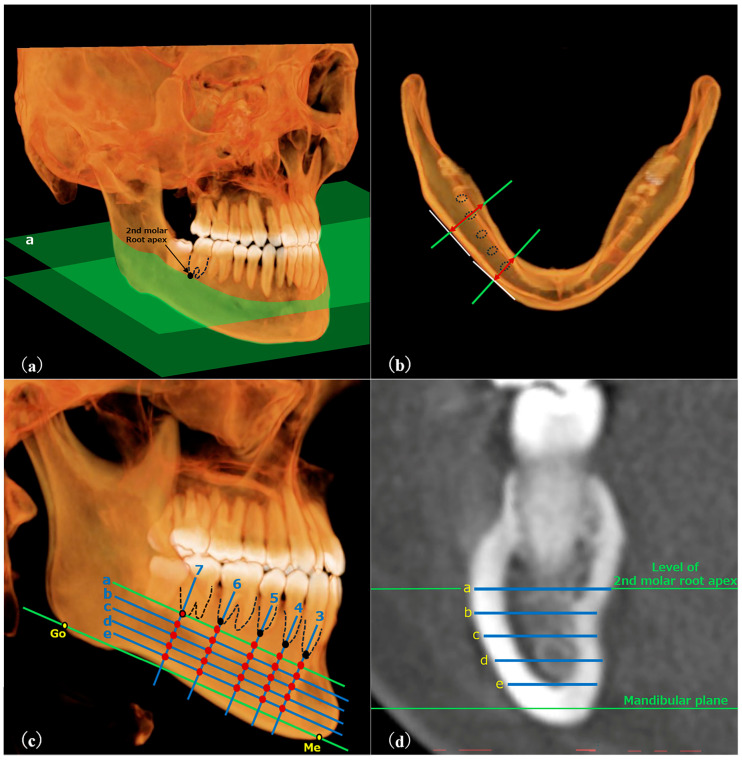
(**a**–**d**) CT images viewed with DICOM viewer. (**a**) Plane “a” was defined as that parallel to the mandibular plane and intersecting the distal root apex of the second molar. (**b**) A plane was established in plane “a” that passed through the root apex (distal apex for molars) and was perpendicular to the tangent of the mandibular buccal surface. (**c**) Five oblique lines were drawn perpendicular to the mandibular plane, intersecting the tooth apices. Four equidistant horizontal lines were drawn from plane “a” to the mandibular plane. Thickness was measured at 25 intersections. (**d**) An example of measurement at a certain cross-section.

RT was measured following the method described by Yeung et al. [12] (Figure 2), while BT was measured using the method described by Swasty et al. [15] for positioning images (Figure 3). Customized settings were used to ensure that measurements were taken at 25 sites per side, similar to RT, using OsiriX MD (Pixmeo, Geneva, Switzerland), with bone mode display density.

**Figure 4:**
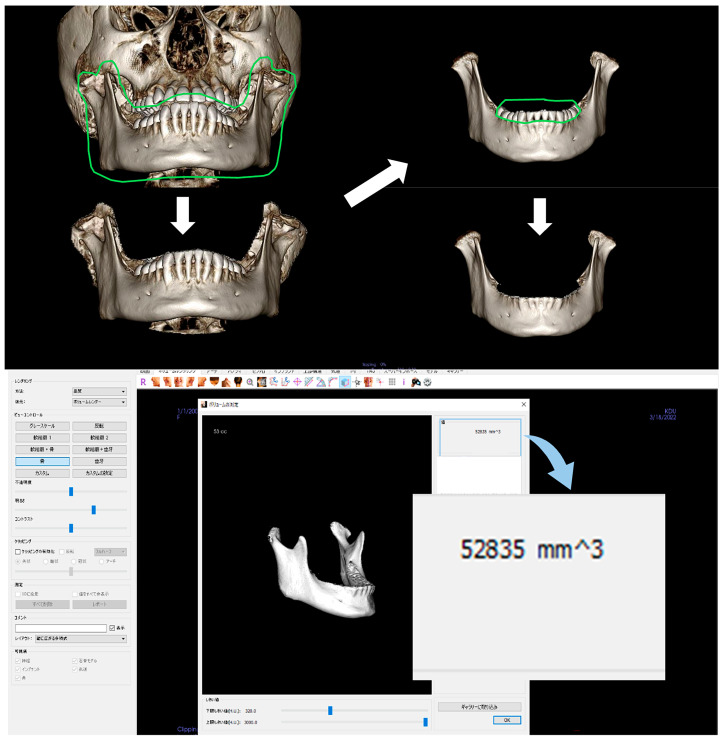
Automatic measurement of the volume of the mandible.

Mandibular volume (V) was measured using the image processing software inVivo6 (Anatomage, San Jose, CA, USA). Trimming was performed in volume-rendering mode to extract only the mandible. Volume measurements were performed using the automatic volume measurement mode. Dental findings were excluded from the trimming range since they significantly affect volume measurements [8]. (Figure 4)

Some of the CT image samples in this study do not contain nasion. Therefore, when measuring ANB using CT, samples that do not include the nasion will either be excluded or measured using cephalometry. Previous research [14] also used cephalometry for cephalometric analysis, rather than CT. We measured ANB using cephalometry because we needed to ensure a sufficient sample size and to ensure the equality of the measurement items.

**Figure 5:**
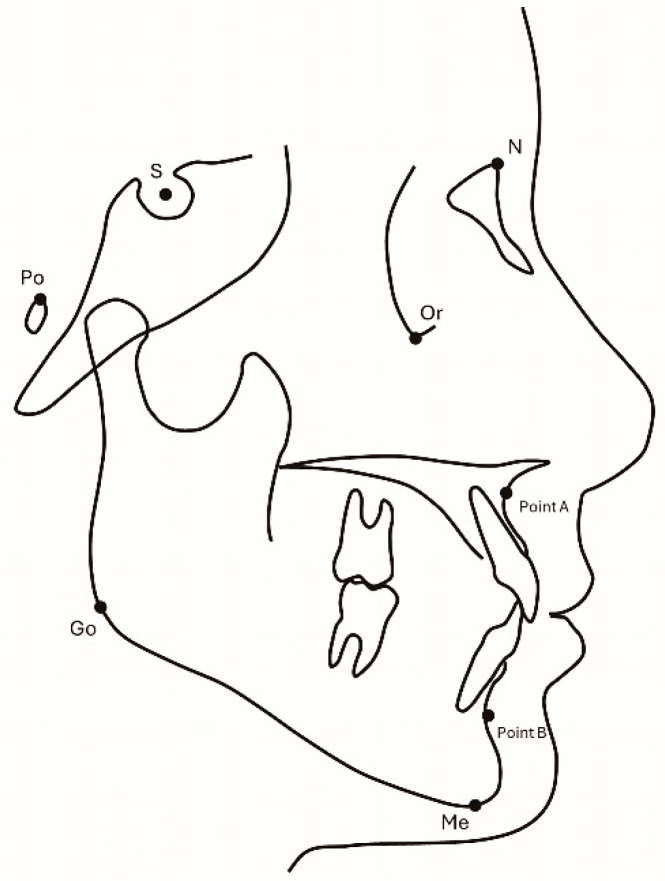
Measurement of lateral cephalograms (ANB [°], FMA [°]). Image credits: Akihiro Tsuboi.

**Figure 6:**
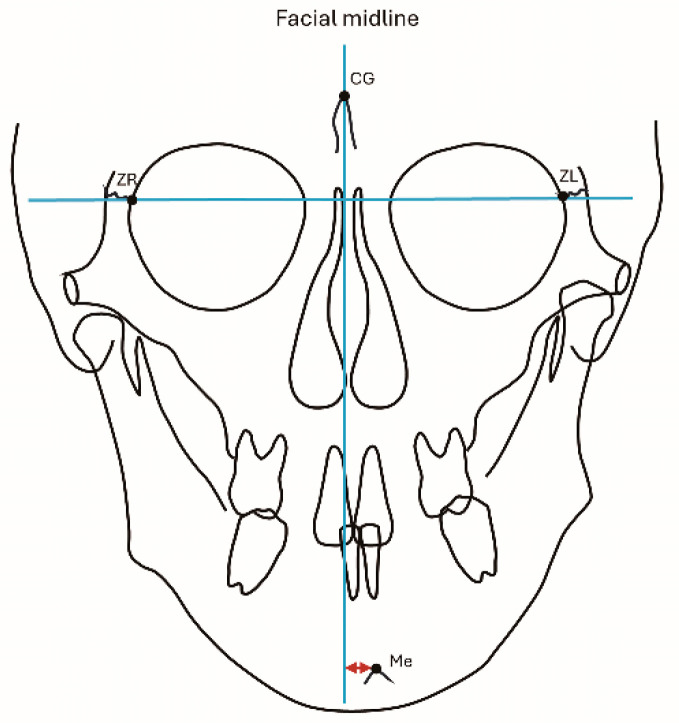
Measurement of Me deviation, representing the vertical distance from the facial midline to Me. Image credits: Akihiro Tsuboi.

Lateral and frontal cephalograms were traced, and measurements of the ANB (°), FMA (°), and SN (mm) were performed using lateral cephalograms (Figure 5). Evaluation of lateral mandibular deviation was conducted using menton deviation (mm). Cases with a lateral deviation of 0–2 mm were considered to have no lateral deviation [16]. Measurements of menton deviation were performed using frontal cephalograms (Figure 6). All measurements were performed by a single researcher. To investigate intra-operator measurement error, 25 cases were randomly selected for remeasurement at 2-week intervals. The measurement error was estimated using Dahlberg’s formula [17].

### 2.2. Statistical Analysis

Multiple linear regression analysis was conducted using the forced entry method, with (BT-RT)/V as the dependent variable and ANB, FMA, age, sex, and type of CT equipment as independent variables to mitigate the influence of mandibular volume on BT-RT. The normality of the residuals was confirmed using the Kolmogorov–Smirnov test, and homoscedasticity in the residuals was assessed using scatter plots.

Statistical analyses were performed using IBM SPSS version 28.0.1 (IBM, Armonk, NY, USA), with a significance level set at 5%.

## 3. Results

The intra-operator measurement error, calculated using Dahlberg’s formula, was observed to be 2.4%, indicating sufficient reproducibility.

This study is exploratory, and due to the lack of similar studies to guide sample size determination, all 224 CT images meeting the inclusion criteria and acquired between February 2017 and March 2023 were included in the analysis. Based on the formula for sample size for detecting correlation coefficients using G*Power (version 3.1.9.7, Franz Faul, Christian Albrechts-Universität, Kiel, Germany), a sample size of 224 was shown to have 80% power to detect a correlation coefficient of approximately 0.18 at a significance level of 5%. Given that many studies on craniofacial morphometrics using CT scans determine sample size based on a correlation coefficient of 0.15, a correlation coefficient of 0.18 is considered a meaningful, and not necessarily small, value in this research context.

The mean, standard error, and maximum and minimum values of cephalogram and CBCT measurement parameters are presented in Table 1. Samples were collected for all anterior–posterior intermaxillary relationships, from skeletal Class II to skeletal Class III. Furthermore, because patients with jaw deformities were also included, the standard deviation of ANB increased due to the inclusion of samples with severe mandibular prognathism and maxillary prognathism. The mean and standard error of measurements for each cross-section of the ramus and body of the mandible are presented in Table 2.

The relationships between RT/V, BT/V, and (BT-RT)/V and the independent variables are shown in Table 3. A significant association was found between RT/V and ANB and gender (*p* < 0.001; ANB: β = 0.303, gender: β = −0.163). For BT/V, a significant association was found with gender (*p* < 0.047; β= −1.994). No significant associations were found between ANB, FMA, and age (*p* < 0.05). For (BT-RT)/V, a significant association was found with ANB (*p* < 0.001; β = −0.248).

Table 4 shows a comparison of the results of multiple linear regression analysis with and without association for each thickness. A significant association was found only for the item of difference in CT divided by SN. For the other items, including the association between the dependent variable and ANB, which is the main topic of this study, there were no differences in the results depending on the method of association.

## 4. Discussion

The study results revealed a negative association between (BT-RT)/V and ANB, contrary to the anticipated growth pattern, which resembled the thinning of stretched pizza dough [12]. Specifically, as anterior mandibular growth progressed, the ramus became notably thinner, while the mandibular body remained unaffected by the degree of anterior growth. This suggests that anterior growth is primarily attributable to the ramus growth, with the mandibular body playing a minimal role. In addition, we observed an association between growth magnitude and mandibular bone thickness. Based on these findings, it is plausible that more pronounced anterior growth leads to a greater difference in thickness between the ramus and body of the mandible. These results enhance our understanding of mandibular growth patterns and may improve the accuracy of mandibular growth prediction.

Tseng et al. [7] previously classified craniofacial morphology horizontally based on ANB as follows: skeletal Class I (0° ≤ ANB < 4°), skeletal Class II (ANB ≥ 4°), and skeletal Class III (ANB < 0°). Upon investigating the thickness of the ramus in skeletal Class II and skeletal Class III, the ramus thickness in skeletal Class III was found to be significantly thinner. The results were replicated in a similar investigation with the sample size increased by more than four times. Additionally, the results were consistent in the comparison of the measured RT between skeletal Class II and skeletal Class III, assessed using the measurement method of Yeung et al. [12].

Seong et al. [18] evaluated the mandibular thickness by dividing the volume of the mandibular body by the length of the mandibular body and reported that skeletal Class III was significantly thinner than skeletal Class I. However, therein, a significant correlation between the ANB and the thickness of the mandibular body was not observed. This discrepancy may be attributed to differences in the interpretation of thickness. In essence, Seong et al. [18] evaluated the cross-sectional area of the mandibular body, which did not eliminate the influence of the height of the mandibular body. In contrast, while this study did not evaluate the entire thickness of the mandibular body, it purely focused on the thickness. Seung et al. [19] investigated the height of the mandibular body on the deviated and non-deviated sides of the mandible and reported that the non-deviated side was significantly higher. Given that there are significant differences in the height of the mandibular body, the results could change when the influence of mandibular body height is excluded.

This study focused on the thickness of the mandible. To investigate the association with each analysis variable, it was necessary to eliminate the influence of individual differences in mandibular size on thickness. Therefore, this study measured the ratio of the difference in thickness to the volume of the mandible, eliminating the influence. The validity of excluding the influence of mandibular size differences on thickness was also examined. As shown in Table 4, only (BT-RT)/SN showed a negative correlation with the difference between CT devices. This indicates that the difference in thickness measured by MDCT is significantly smaller. When comparing the measurement devices used in this study, MDCT has a larger voxel size and poorer spatial resolution, so it is less clear in its depiction of the cortical bone surface. Because the points on the cortical bone surface are plotted based on the visual judgment of the measurer, it is possible that the MDCT images were measured to be shorter than their actual thickness. On the other hand, in this study, we used aluminum square bars to evaluate the measurement error between CT devices, and confirmed that the error was sufficiently small for automatic volume measurement, but this discrepancy can be interpreted as being due to the presence or absence of visual judgment by the measurer. The reason why a significant correlation was only seen when divided by (BT-RT)/SN is as follows. Because the SN value is measured using cephalometry and not computed tomography, dividing by the SN value may not cancel out the measurement error described above. In addition, although BT-RT does not show a significant correlation in the CT difference item, when compared with (BT-RT)/V and RT/BT, which are ratios of values measured on CT images, the p-value is very small. From the above, it is thought that the method of dividing by the volume of the mandible and the method of taking the ratio of the thickness of the ramus to the thickness of the mandible body may be able to more accurately correct the measurement results.

The mandible undergoes both endochondral and intramembranous ossification, representing dual ossification patterns [20]. The observed relationship between the anteroposterior relationship and thickness differences in our study may stem from differences in ossification patterns between the ramus and the body of the mandible. Lee et al. [21] discovered the mandibular primary growth center in the fetal mandible prenatally, located at the apical area of the deciduous first molar. They further observed that a conical region extending from the mandibular primary growth center to the mandibular condyle was formed through endochondral ossification, whereas the remaining areas underwent intramembranous ossification. Until recently, endochondral and intramembranous ossifications were considered independent ossification patterns. However, Tsukasaki et al. [22] revealed that signaling molecules derived from periosteal stem cells promote endochondral ossification. This suggests that the anteroposterior growth of the ramus may increase with the promotion of intramembranous ossification. If intramembranous ossification proceeds radially at a consistent rate from the mandibular primary growth center, it is possible that as the anteroposterior growth of the ramus becomes more pronounced, the portion formed by intramembranous ossification of the ramus may become thinner.

Lee et al. [21] reported similar growth rates from the mandibular primary growth center toward both the gonion and mandibular symphysis, but a significantly higher rate toward the condylar head. This suggests that the growth rate in the direction of the mandibular ramus was higher than the anteroposterior growth rate of the mandibular body. Additionally, Bareggi et al. [23] conducted a study on the early development of the fetal mandible and observed that the growth rate of the ramus in terms of length and height was faster than that of the mandibular body. Even during the fetal period, the mandible exhibits differences in growth between the ramus and body, implying that changes in thickness associated with the growth of each region may not be uniform. Remy et al. [24] reported that the mandible undergoes its most significant growth during the first 5 years of life. Additionally, Hutchinson et al. [25] reported that the shape and size of the mandible continue to change until the age of 3 years. These findings suggest that the morphological features of the mandible observed in this study were already present in pediatric mandibles at the initiation of orthodontic treatment during the mixed dentition period. In the future, longitudinal studies with samples from patients who have undergone orthodontic treatment during the mixed dentition period and followed up with adult orthodontic treatment could aid in predicting growth patterns. Specifically, CT scans taken during orthodontic treatment in the mixed dentition period could be used to measure the thickness of the ramus and body of the mandible and ANB could be evaluated using lateral cephalograms obtained during adult orthodontic treatment. Investigating the correlation between these measurements could facilitate future mandibular growth prediction from CT scans obtained during orthodontic treatment in the mixed dentition period.

Furthermore, while growth and sex hormones continue to influence mandibular growth after puberty, thyroid hormone is crucial for mandibular formation before puberty [26]. Kesterke et al. [27] revealed that variations in thyroid hormone secretion levels affect mandibular growth in mouse embryos. By investigating the aforementioned morphological characteristics of the mandible in early adolescence before puberty, it may be possible to enhance predictions of future mandibular growth.

### Limitation

There is some concern about the wide age range in this study, but Dong et al. [28] also collected samples from the same age range as this study.

Ideally, we would have only included samples taken using the same equipment to account for differences in CT scans, but if we had limited ourselves to the same equipment, the sample size would have become too small. Therefore, in this study, we decided that we could deal with this by including differences in CT equipment as explanatory variables, and decided to include images taken using different equipment in the sample.

Furthermore, the proportion of female patients was high in the sample (156 females, 68 males). We believe that there is no bias in this study because the samples are all CT images taken within a certain period of time that meet the conditions. The reason for the gender imbalance is probably because there are more female patients than male patients who wish to receive orthodontic treatment at our hospital. When the difference between the thickness of the body and the thickness of the mandibular ramus, which is the main topic of this study, is divided by the volume of the mandible, the statistical results show no significant correlation between the sexes (*p* = 0.790), so it is thought that there is no bias in the sample, but the statistical results for each thickness show a significant correlation between the sexes (*p* = 0.014, 0.047). This suggests that the bias in the male–female ratio may have affected these results. In future research, it will be necessary to improve the generalizability of the results by ensuring a more balanced male–female ratio in the sample. The ratios of RT/V, BT/V, and (BT-RT)/V showed a negative correlation with gender. When comparing the items in Table 4 by gender, only the significant negative correlation was observed when divided by the volume of the mandible. From this, it is thought that the volume of the mandible is significantly smaller in women, and there is a possibility that there is no gender difference in the thickness itself. This interpretation is limited due to the bias in the sample.

## 5. Conclusions

The variation in bone thickness associated with anterior mandibular growth appears to vary by location. The change in thickness suggests that the difference in thickness between the ramus and body enlarges with increasing mandibular protrusion. This discrepancy in thickness is primarily attributed to the ramus, suggesting that the thickness of the body part remains unaffected by anterior growth. The morphological characteristics of the mandible discovered in this study may be used as a basis for predicting mandibular growth in the diagnosis of pediatric orthodontics in the future. In order to do this, it is necessary to conduct a longitudinal study by investigating the morphological characteristics of the mandible found in this study in patients in the mixed dentition period, and there is the possibility that a growth prediction model can be created based on the morphological characteristics of the mandible in the mixed dentition period of the same patient and the anteroposterior intermaxillary relationship after reaching adulthood.

## Figures and Tables

**Table 1:** Means and standard deviations (SDs) of the measurements from computed tomography (CT) and lateral cephalogram analysis.

	Male (n = 68)		Female (n = 156)		Total (n = 224)	
	Mean ± SD	Range	Mean ± SD	Range	Mean ± SD	Range
Age, y	26.6 ± 0.8	18.3–60.0	29.1 ± 0.4	18.0–65.1	28.3 ± 0.9	18.0–65.1
Mandibular volume, mm^3^	53,400.0 ± 1427.0	38,769.0–71,077.0	45,981.4 ± 671.3	31,016.0–67,992.0	47,683.5 ± 8547.4	31,016.0–71,077.0
Class I, mm^3^	51,801.4 ± 9932.3	31,036.0– 71,079.0	48,999.8 ± 9784.9	34,164.0– 66,303.0	50,812.9 ± 9953.3	31,036.0– 71,079.0
Class II, mm^3^	49,640.6 ± 2586.1	38,769.0–68,792.0	46,000.0 ± 840.4	31,199.0–63,561.0	46,520.0 ± 814.9	31,199.0–68,792.0
Class III, mm^3^	55,071.9 ± 1640.8	39,204.0–71,077.0	45,958.8 ± 1089.8	31,016.0–67,992.0	48.819.9 ± 1012.4	31,016.0–71,077.0
hypodivergent, mm^3^	51,208.5 ± 2081.6	38,769.0–68,792.0	45,104.0 ± 845.5	31,016.0–67,992.0	46,088.6 ± 7852.2	31,016.0–68,792.0
normodivergent, mm^3^	54,560.3 ± 2750.0	39,204.0–71,077.0	47,056.6 ± 1230.2	34,143.0–66,456.0	49,038.7 ± 8986.2	34,143.0–71,077.0
hyperdivergent, mm^3^	55,065.5 ± 2609.8	40,154.0–65,490.0	48,239.4 ± 2705.6	34,163.0–65,571.0	51,342.2 ± 9343.7	34,163.0–65,571.0
ANB, °	−1.2 ± 0.9	−9.0–7.5	2.5 ± 0.5	−12.0–15.1	1.7 ± 5.4	−12.0–15.1
Mandibular plane angle, °	28.4 ± 1.3	15.1–56.0	32.6 ± 0.7	16.0–67.0	31.6 ± 8.2	15.1–67.0

**Table 2:** Mean thicknesses of subjective mandibles: ramus and mandibular body (N = 224).

	Mean ± SD	Range
Thickness of the ramus, mm (25 points)	7.26 ± 1.11	1.41 to 20.90
Thickness of the ramus (5 points per row/column)		
Row 1, mm	7.74 ± 0.11	2.04 to 20.90
Row 2, mm	8.31 ± 0.09	1.24 to 16.40
Row 3, mm	7.68 ± 0.09	1.41–13.50
Row 4, mm	6.20 ± 0.06	1.89–13.14
Row 5, mm	5.94 ± 0.05	2.89–11.98
Column A, mm	4.99 ± 0.05	1.24–14.40
Column B, mm	6.18 ± 0.06	2.10–12.80
Column C, mm	7.45 ± 0.06	2.92–14.40
Column D, mm	8.39 ± 0.08	2.95–15.80
Column E, mm	8.86 ± 0.09	2.89–20.90
Thickness of the mandibular body (25 points)	10.32 ± 0.89	0.47–16.50
Thickness of the mandibular body (5 points per row/column)		
Row a, mm	10.87 ± 0.07	5.30–16.50
Row b, mm	10.64 ± 0.06	5.30–16.50
Row c, mm	10.45 ± 0.05	5.35–14.70
Row d, mm	10.39 ± 0.12	1.11–10.60
Row e, mm	9.15 ± 0.04	0.47–12.70
Column 7, mm	11.30 ± 0.13	6.60–10.60
Column 6, mm	10.86 ± 0.06	0.47–16.30
Column 5, mm	10.08 ± 0.05	1.11–15.30
Column 4, mm	9.75 ± 0.04	5.35–14.30
Column 3, mm	9.50 ± 0.05	5.30–13.20

SD, standard deviation.

**Table 3:** Association tests using multiple linear regression analyses.

Dependent Variable	Independent Variables	B	SE	β	*p*	Pearson’s *r*
RT/V						
	ANB, °	0.00000230	0.00000057	0.303	<0.001 ***	0.317
	FMA, °	0.00000002	0.00000032	0.040	0.566	0.186
	Age	−0.00000003	0.00000021	−0.095	0.135	−0.045
	Sex (Female = 0, Men = 1)	−0.00000130	0.00000526	−0.163	0.014 **	−0.238
	CT (CBCT = 0, MDCT = 1)	0.00000640	0.00000511	0.085	0.215	0.01
BT/V						
	ANB, °	0.00000070	0.00000078	0.853	0.395	0.075
	FMA, °	−0.00000020	0.00000044	−0.383	0.702	0.029
	Age	0.00000003	0.00000029	0.118	0.906	−0.027
	Sex (Female = 0, Men = 1)	−0.00001430	0.00000717	−1.994	0.047 **	−0.155
	CT (CBCT = 0, MDCT = 1)	0.00000476	0.00000696	0.684	0.684	0.037
(BT-RT)/V						
	ANB, °	−0.00000167	0.00000052	−0.248	<0.001 ***	−0.254
	FMA, °	−0.00000035	0.00000029	−0.087	0.226	−0.17
	Age	0.00000035	0.00000019	0.120	0.067	0.091
	Sex (Female = 0, Men = 1)	−0.00000127	0.00000475	−0.018	0.790	−0.046
	CT (CBCT = 0, MDCT = 1)	−0.00000159	0.00000461	−0.024	0.731	0.043

SE, standard error; RT, ramus thickness; BT, mandibular body thickness; V, mandibular volume; CT, computed tomography; CBCT, cone-beam CT; MDCT, multi-detector CT; FMA, Frankfort-mandibular plane angle. ** *p* < 0.05, *** *p* < 0.001.

**Table 4:** Comparison of multiple linear regression analysis results for different thickness correction methods.

Dependent Variable	Independent Variables	B	SE	β	*p*
BT-RT					
	ANB, °	−0.082	0.021	−0.295	<0.001 ***
	FMA, °	−0.017	0.011	−0.103	0.135
	Age	0.015	0.008	0.121	0.056
	Sex (Female = 0, Men = 1)	0.326	0.189	0.114	0.085
	CT (CBCT = 0, MDCT = 1)	-0.290	0.183	−0.108	0.114
(BT-RT)/SN					
	ANB, °	−0.0012	0.000320	−0.298	<0.001 ***
	FMA, °	−0.0002	0.000180	−0.097	0.166
	Age	0.0032	0.000120	0.068	0.288
	Sex (Female = 0, Men = 1)	0.0020	0.002890	0.049	0.460
	CT (CBCT = 0, MDCT = 1)	-0.0063	0.002800	−0.155	0.027 **
BT/RT					
	ANB, °	−0.015	0.003	−0.338	<0.001 ***
	FMA, °	−0.002	0.002	−0.068	0.320
	Age	0.003	0.001	0.158	0.013 **
	Sex (Female = 0, Men = 1)	0.036	0.031	0.077	0.239
	CT (CBCT = 0, MDCT = 1)	-0.008	0.030	−0.019	0.782
(BT-RT)/V					
	ANB, °	−0.00000167	0.00000052	−0.248	<0.001 ***
	FMA, °	−0.00000035	0.00000029	−0.087	0.226
	Age	0.00000035	0.00000019	0.120	0.067
	Sex (Female = 0, Men = 1)	−0.00000127	0.00000475	−0.018	0.790
	CT (CBCT = 0, MDCT = 1)	−0.00000159	0.00000461	−0.024	0.731

SE, standard error; RT, ramus thickness; BT, mandibular body thickness; V, mandibular volume; CT, computed tomography; CBCT, cone-beam CT; MDCT, multi-detector CT; FMA, Frankfort-mandibular plane angle. ** *p* < 0.05, *** *p* < 0.001.

## Data Availability

The data presented in this study are available from the corresponding author upon request.
